# The importance of EGFR mutation testing in squamous cell carcinoma or non-small cell carcinoma favor squamous cell carcinoma diagnosed from small lung biopsies

**DOI:** 10.1186/s13000-019-0840-2

**Published:** 2019-06-21

**Authors:** Hsiang-Ling Ho, Hua-Lin Kao, Yi-Chen Yeh, Teh-Ying Chou

**Affiliations:** 1 Division of Molecular Pathology, Department of Pathology and Laboratory Medicine, Taipei Veterans General Hospital, Taipei, Taiwan; 2 Department of Biotechnology and Laboratory Science in Medicine, National Yang-Ming University, Taipei, Taiwan; 3 Institute of Clinical Medicine, National Yang-Ming University, Taipei, Taiwan; 4 Department of Pathology, Taipei Medical University Hospital, Taipei, Taiwan

**Keywords:** Lung cancer, EGFR mutation, Squamous cell carcinoma, Adenosquamous carcinoma, Small biopsy

## Abstract

**Background:**

Adenosquamous carcinoma (ADSC) of the lung, a rare but aggressive subtype of non-small cell lung cancer (NSCLC), is defined as a carcinoma containing components of adenocarcinoma (ADC) and squamous cell carcinoma (SqCC). Mutations of epidermal growth factor receptor (EGFR) are found at a frequency of 15 to 44% in Asian ADSC, and EGFR tyrosine kinase inhibitors (EGFR-TKIs) are a more effective treatment for EGFR-mutated ADSC compared to chemotherapy. However, ADSC in small lung biopsies could be misdiagnosed as SqCC or non-small cell carcinoma (NSCC) favor SqCC due to undersampling, which may result in neglecting of *EGFR* mutation testing and affecting patients’ clinical management, particularly in Asian patients that relatively have high prevalence of *EGFR* mutation.

**Methods:**

A total of 148 small lung biopsy cases with pathological diagnosis of SqCC or NSCC favor SqCC were retrospectively enrolled. The frequency of *EGFR* mutations and the correlation between patients’ *EGFR* mutation status and clinicopathological characteristics were evaluated.

**Results:**

*EGFR* mutations were found in 8.8% (13 /148) of all cases with 5.2% (7/135) in SqCC and 46.2% (6/13) in NSCC favor SqCC. There were 7 (53.8%) L858R mutation, 4 (30.8%) exon 19 deletions, and 2 (15.4%) cases with coexistent L858R and T790 M mutations. Multivariate analysis showed that *EGFR* mutations were more prevalent in never-smokers (83.3% versus 16.7%, *p* = 0.006) and patients diagnosed as NSCC favor SqCC (46.2% versus 5.2%, *p* = 0.001). Moreover, 75% (3/4) of *EGFR* mutation-positive cases with subsequent surgical resection or rebiopsy were further diagnosed as ADSC.

**Conclusions:**

*EGFR* mutation testing should be performed in Asian patients with SqCC diagnosed from small lung biopsies, especially in never-smokers and patients with diagnosis of NSCC favor SqCC, which have a high probability of being ADSC.

## Background

Lung cancer is the most common cause of cancer-related death worldwide. Non-small cell lung cancer (NSCLC), accounting for approximately 80–85% of all the cases, is further divided into major histological subtypes including adenocarcinoma (ADC), squamous cell carcinoma (SqCC), large cell carcinoma (LCC), and other rare tumors including adenosquamous carcinoma (ADSC) [1]. ADSC is a rare and mixed-type NSCLC, counting for only 0.4 to 4% of all the cases. According to the World Health Organization (WHO) Classification of Lung Tumors, ADSC is defined as “a carcinoma showing components of both SqCC and ADC, with each comprising at least 10% of the tumor” [2]. ADSC has a more aggressive behavior and worse prognosis than ADC and SqCC [3, 4]. The treatment of early-stage ADSC mainly includes surgery, supplemented by chemotherapy, and other comprehensive treatment, but the curative effect is not satisfied [5]. The poor prognosis of ADSC has raised the demand for more effective treatment approaches such as targeted therapy. Recently, the development of targeted therapy toward oncogenic “driver mutations” has revolutionized the clinical management of patients with NSCLC including ADSC [6, 7].

Epidermal growth factor receptor (EGFR) mutation is the most well-characterized driver mutation in NSCLC, and is more prevalent in Asian population, never smokers, and patients with ADC subtype [8]. More than 60% of Asian ADC patients were found to have *EGFR* mutations [9]. EGFR tyrosine kinase inhibitors (EGFR-TKIs), giving higher response rates, longer progression-free survival and better quality of life compared to conventional chemotherapy, are now recommended as the first-line treatment in patients with advanced *EGFR* activating mutation-positive NSCLC [10–13]. Currently, studies demonstrated that *EGFR* mutations occur at a frequency of 15 to 44% in Asian ADSC and are mainly in female and never smokers; patients with ADSC showed a similar benefit from EGFR-TKI treatment compared to those with ADC, revealing that EGFR-TKI may be a therapeutic option for ADSC patients [14–18]. In view of this, the current guidelines recommend that *EGFR* mutation testing should be performed in NSCLC patients with ADC or mixed-type tumors containing an adenocarcinomatous component, for example, ADSC, and is not necessary for patients with tumors lack of any adenocarcinomatous component, such as pure SqCC or pure LCC [19, 20].

In clinical practice, accurate diagnosis of ADSC can only be made in resection specimens, because a minimum of 10% of each ADC or SqCC component is required. However, approximately 70% of lung cancer patients are diagnosed at advanced stages that are not amenable to surgical resection and small biopsies are the major tissue resources for pathological diagnosis and subtyping [21, 22]. Diagnosis of ADSC before surgical resection is not possible, since tumors present in small biopsy specimens may come from either ADC or SqCC component only. In the majority of cases, ADSC in small biopsy specimens is misdiagnosed as SqCC or poorly differentiated SqCC referring to non-small cell carcinoma (NSCC) favor SqCC due to biased sampling of the lesion, which may cause the neglect of *EGFR* mutation testing in these cases. A previous report by Rekhtman et al. also suggested that *EGFR* or *KRAS* mutations do not occur in pure pulmonary SqCC, and the occasional detection of these mutations may come from the challenge of ADSC diagnosis in small biopsies [23]. Furthermore, two NSCLC cases, possibly ADSC, presented by Saini et al. were initially diagnosed as SqCC in small biopsies, positive for *EGFR* mutations and benefitted with oral EGFR-TKI treatment [24]. The lack of *EGFR* mutation testing in patients who are diagnosed as SqCC from small biopsies may influence the treatment strategies for patients, especially in Asian population which has a high frequency of *EGFR* mutations. For this reason, we conducted an Asian retrospective study investigating the frequency and clinicopathological association of *EGFR* mutations in patients diagnosed as SqCC in small lung biopsies to evaluate the necessity and clinical impact of *EGFR* mutation testing on possibly under-sampled ADSC.

## Methods

### Patients

From 2007 to 2016 at Taipei Veterans General Hospital, patients with a diagnosis of SqCC or NSCC favor SqCC derived from small lung biopsies and without previous history of any carcinoma elsewhere in the body were evaluated in this retrospective study. Each case was reviewed by two independent pathologists, and all final diagnoses were made according to the 2015 WHO classification of lung cancer in small biopsies and cytology [2, 25]. H&E stained slides were firstly used for standard morphological diagnosis. Tumors with SqCC morphological features, such as keratinization, intercellular bridge, and keratin pearl formation were diagnosed as SqCC. For those cases composed of poorly differentiated NSCLC without definite SqCC morphology, immunohistochemistry (IHC) was used to assist the pathological diagnosis. Cases positive for SqCC markers (p40 or CK5/6) with at least moderate to diffuse staining pattern and strong intensity, and negative for ADC markers (TTF-1 and/or mucin) were classified as NSCC favor SqCC. Cases with final diagnosis of SqCC or NSCC favor SqCC determined from small biopsies were further analyzed for *EGFR* mutation status and those without additional tissue sections for molecular tests were excluded in this study. This retrospective study was approved by the Institutional Review Board of Taipei Veterans General Hospital (2017–05-008CC).

### Scoring of immunohistochemistry

The tissue sections were immunostained with commercially available antibodies including thyroid transcription factor-1 (TTF-1) (clone 8G7G3/1, Dako), p40 (clone BC28, Biocare Medical) and CK5/6 (clone D5/16 B4, Dako) on a Leica BOND-MAX system (Leica Microsystems, Newcastle, UK). Tumors completely absent of TTF-1 staining were considered as TTF-1 negative. For p40 or CK5/6 staining, the moderate staining pattern was defined as 10 to 50% of tumor cells showing immunoreactivity, while the diffuse staining pattern was defined as more than 50% of tumor cells with immunoreactivity. Cases with staining intensity similar to internal controls represented by bronchial/bronchiolar basal cell layer were recognized as strong intensity.

### EGFR mutation analysis

H&E stained tissue slides were reviewed by pathologists to select the tumor areas and the selected areas were microdissected manually from the corresponding ones in the consecutive tissue sections and, after deparaffinization, were subjected to genomic DNA extraction. Briefly, the microdissected tumor tissue was transferred to an eppendorf tube containing Proteinase K solution (PicoPure™ DNA Extraction Kit, Applied Biosystems™). The tube was then incubated at 56 °C for 16 h, followed by an inactivation step at 95 °C for 10 min. The Proteinase K digested extract was adequate for molecular tests. Since *EGFR* exon 19 deletions and L858R mutation are the most common types of *EGFR* mutations, together accounting for approximately 90% of TKI-sensitive mutations, TaqMan EGFR Exon 19 Deletions Assay, L858R Mutation Detection Assay and Reference Assay (Invitrogen Life Technologies Inc., Waltham, MA, USA) were used to examine *EGFR* mutations according to the manufacturer’s instruction. Real-time PCR was performed using the StepOnePlus™ Real-Time PCR System (Applied Biosystems). The mutation status of a sample was determined by using the parameter described previously [26]. Samples were considered as mutation positive if a specific sigmoid curve was observed with Ct of mutation reaction ≤37 and/or ΔCt value ≤7.

### Statistical analysis

The correlation between patients’ clinicopathological features and *EGFR* mutation status was assessed using chi-square test or independent sample t-test. For multivariate analysis, logistic regression model was used. All analyses were performed using the Statistical Product and Service Solutions program 18.0 for windows (SPSS, Chicago, IL). *p* value below 0.05 was considered to indicate statistical significance.

## Results

### Patients’ clinical characteristics

One hundred and forty-eight patients diagnosed as SqCC or NSCC favor SqCC from small biopsies were enrolled in this study. The patients’ clinicopathological characteristics were summarized in Table 1. There were 78 (52.7%) male and 70 (47.3%) female patients ranging from 41 to 93 years (median age, 73 years). Among them, 135 (91.2%) were diagnosed as SqCC based on standard H&E morphological criteria, while 13 (8.8%) were NSCC favor SqCC based on IHC results. Of 128 patients with known smoking status, 39 (30.5%) were never smokers and 89 (69.5%) were current or ever smokers. For methodologies of obtaining biopsy specimens, 72 (56.7%) were through bronchoscopy, 40 (31.5%) computed tomography (CT) guided-biopsy, and 15 (11.8%) sono-guided biopsy. The average of biopsy pieces per sample was 4.4 ranging from 1 to 13 pieces, and the mean size of samples was 6.6 mm in the largest diameter.

**Table 1:** Comparison of clinicopathological characteristics between *EGFR* mutation and *EGFR* wild-type cases with SqCC or NSCC favor SqCC diagnosed from small biopsies

Characteristics	Total		EGFR mutation		EGFR wild-type		Univariate analysis		Multivariate analysis	
	No.	%	No.	%	No.	%				
Number	148	100.0 (148/148)	13	100.0 (13/13)	135	100.0 (135/135)				
Age(y)							*P = 0.527*		*P = 0.810*	
Median	70.2 ± 12.4		67.5 ± 16.0		70.4 ± 12.1					
Range	41–93		44–86		41–93					
Sex							P = 0.039*		*P = 0.841*	
Male	78	52.7 (78/148)	3	23.1 (3/13)	75	55.6 (75/135)				
Female	70	47.3 (70/148)	10	76.9 (10/13)	60	44.4 (60/135)				
Smoking status							P < 0.0001*		P = 0.006*	
Never smoker	39	30.5 (39/128)	10	83.3 (10/12)	29	25.0 (29/116)				
Smoker	89	69.5 (89/128)	2	16.7 (2/12)	87	75.0 (87/116)				
Unknown	20		1		19					
Biopsy method							*P = 0.826*			
Bronchoscopic	72	56.7 (72/127)	6	50.0 (6/12)	66	57.4 (66/115)				
CT-guided	40	31.5 (40/127)	4	33.3 (4/12)	36	31.3 (36/115)				
Sono-guided	15	11.8 (15/127)	2	16.7 (2/12)	13	11.3 (13/115)				
Unknown	21		1		20					
Biopsy tissue										
Average pieces	4.4 ± 2.0		4.4 ± 2.2		4.4 ± 2.0		*P = 0.925*			
Mean size (mm)	6.6 ± 4.0		6.6 ± 3.3		6.6 ± 4.0		*P = 0.964*			
Histological subtype							P < 0.0001*		P = 0.001*	
SqCC	135	91.2 (135/148)	7	53.8 (7/13)	128	94.8 (128/135)				
NSCC favor SqCC	13	8.8 (13/148)	6	46.2 (6/13)	7	5.2 (7/135)				

*Statistically significant

### EGFR mutation status

As shown in Table 1, *EGFR* mutations were found in 8.8% (13/148) of all cases with 7 (53.8%) having L858R mutation, 4 (30.8%) exon 19 deletions, and 2 (15.4%) coexistence of L858R and T790 M mutations. Among *EGFR* mutation-positive cases, 76.9% (10/13) were female, 83.3% (10/12) were never smokers, and 46.2% (6/13) were NSCC favor SqCC. In univariate analysis, *EGFR* mutations were more frequently observed in female (76.9% versus 23.1%, *p* = 0.039), never smokers (83.3% versus 16.7%, *p* < 0.0001), and patients diagnosed as NSCC favor SqCC (46.2% versus 5.1%, p < 0.0001). Neither age, nor biopsy methods, nor the pieces and size of biopsy samples was significantly associated with *EGFR* mutations. The multivariate analysis showed that *EGFR* mutations were only significantly associated with never smokers (*p* = 0.006) and patients with a diagnosis of NSCC favor SqCC (*p* = 0.001).

### Pathological features in EGFR mutation-positive SqCC diagnosed from small biopsies

**Fig. 1:**
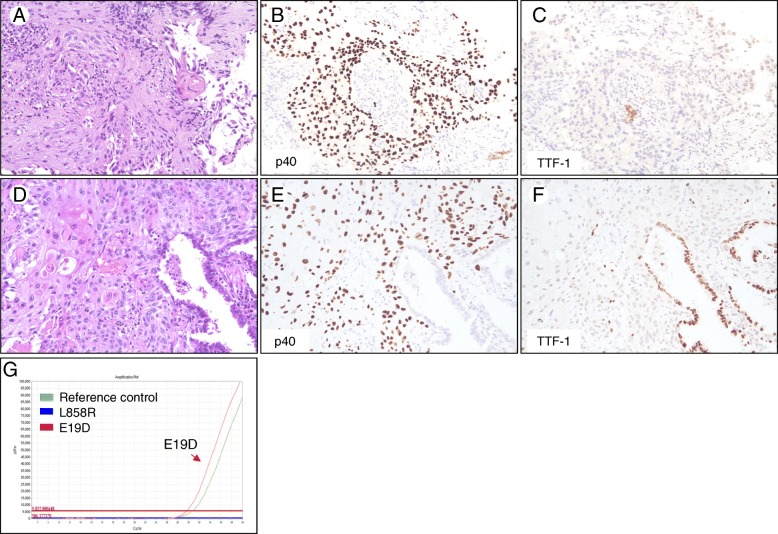
A representative case showing moderately differentiated SqCC in bronchoscopic biopsy diagnosed as adenosquamous carcinoma after surgical resection. **a** Bronchoscopic biopsy specimen displaying classical SqCC morphology with keratin pearl formation. **b**, **c** Tumor cells in the biopsy specimen positive for p40 and negative for TTF-1 staining. **d** Subsequent surgical resection demonstrating a diagnosis of ADSC. **e**, **f** The squamous carcinoma component immunoreactive with p40 and non-reactive with TTF-1, while the adenocarcinoma component immunoreactive with TTF-1 and non-reactive with p40. **g** Real-time PCR analysis of the biopsy specimen positive for *EGFR* exon 19 deletions

**Fig. 2:**
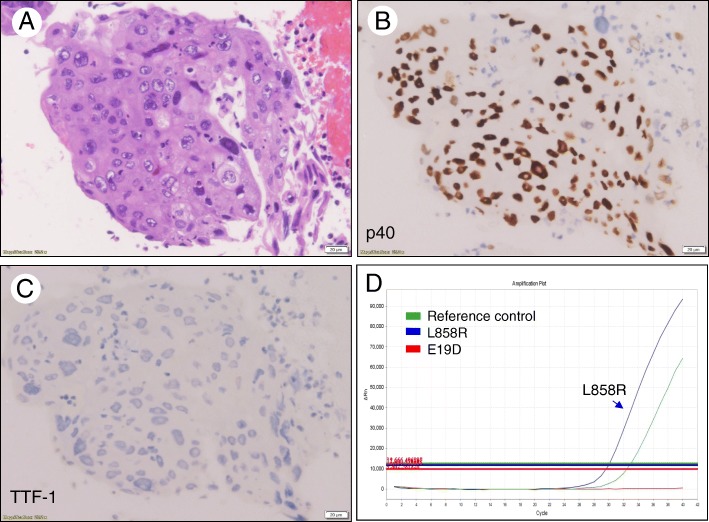
A representative case showing poorly differentiated NSCC favor SqCC in bronchoscopic biopsy positive for *EGFR* L858R mutation. **a** Poorly differentiated carcinoma with distinct cell border and abundant cytoplasm. **b**, **c** Tumor cells with diffuse and strong staining of p40 and negative staining of TTF-1. **d** Real-time PCR analysis positive for *EGFR* L858R mutation

As shown in Table 2, among 13 *EGFR* mutation-positive cases, 7 were diagnosed as SqCC based on standard H&E morphological criteria with keratinization, intercellular bridge or keratin pearl formation. These cases showed strong and diffuse nuclear staining of p40, and negative staining of TTF-1 in IHC analysis. A representative case was shown in Fig. 1. There were 6 cases initially diagnosed as NSCLC-not otherwise specified (NOS) based on H&E staining, showing poorly-differentiated NSCC without glandular, squamous, or neuroendocrine differentiation. These cases were finally diagnosed as NSCC favor SqCC based on IHC results showing moderate to diffuse staining and moderate or strong intensity of p40 or CK5/6, and negative staining of TTF-1 and mucin. A representative case was illustrated in Fig. 2. Moreover, 4 of 13 *EGFR* mutation-positive cases had additional follow-up tumor tissues due to further surgical resection or rebiopsy after initial diagnosis. Three of them had their subsequent lobectomy specimens showing ADSC and one showed exclusively SqCC morphology in the follow-up lung biopsy (Table 2).

**Table 2:** Clinicopathological summary for *EGFR* mutation-positive patients with SqCC or NSCC favor SqCC diagnosed from small biopsies

Patient	Age	Sex	Smoking Status	Mutation	Histologic subtype	ADC marker	SqCC marker	Additional specimens
1	44	F	Never smoker	E19D	SqCC	n.a.	n.a.	Subsequent LUL lobectomy with diagnosis as ADSC
2	57	F	Never smoker	E19D	SqCC	TTF1 (−)	CK5/6 (+); diffuse	Another RLL biopsy with diagnosis as SqCC
3	59	F	Never smoker	L858R/T790 M	SqCC	TTF1 (−)	CK5/6 (+); diffuse	n.a.
4	51	F	Never smoker	L858R/T790 M	SqCC	n.a.	n.a.	n.a.
5	81	F	Smoker	E19D	NSCC favor SqCC	TTF1 (−)	p40 (+); diffuse	n.a.
6	58	F	Never smoker	E19D	NSCC favor SqCC	TTF1 (−)	p40 (+); diffuse	Subsequent RUL lobectomy with diagnosis as ADSC
7	76	F	Never smoker	L858R	NSCC favor SqCC	TTF1 (−)	p40 (+); diffuse	n.a.
8	63	F	Never smoker	L858R	SqCC	TTF1 (−)	p40 (+); diffuse	n.a.
9	84	F	Unknown	L858R	SqCC	TTF1 (−)	p40 (+); diffuse	n.a.
10	74	F	Never smoker	L858R	SqCC	TTF1 (−)	p40 (+); diffuse	Subsequent RUL lobectomy with diagnosis as ADSC
11	86	M	Smoker	L858R	NSCC favor SqCC	TTF1 (−)	p40 (+); moderate	n.a.
12	81	M	Never smoker	L858R	NSCC favor SqCC	TTF1 (−)	p40 (+); moderate	n.a.
13	81	M	Never smoker	L858R	NSCC favor SqCC	TTF1 (−)	p40 (+); moderate	n.a.

*n.a* not available

## Discussion

Recent advances in targeted therapy have dramatically changed the clinical management of lung cancer patients and the molecular testing for driver mutations is now routinely used to predict patients’ treatment response. The current guideline of CAP/IASLC/AMP suggests that testing for *EGFR* mutations and anaplastic lymphoma kinase (*ALK*) translocations should be recommended for adenocarcinomas and mixed lung cancers with an adenocarcinomatous component, regardless of histologic grade, and are not recommended in lung cancers that lack any adenocarcinoma component, such as “pure” SqCC, “pure” SCLC, or large cell carcinomas lacking any IHC evidence of adenocarcinoma differentiation [20]. In the era of precision medicine, accurate pathologic classification of NSCLC is mandatory for tailoring patients to specific molecular diagnostic testing. However, most of advanced lung cancers are diagnosed via tumor biopsies, which increases the difficulty in making a correct diagnosis, particularly in tumors without clear differentiation or in mixed-type tumors, such as ADSC.

Our study focuses on investigating the impact of *EGFR* mutation testing on ADSC in small biopsy diagnostics, especially in Asian patients that have high incidence of *EGFR* mutations. The misdiagnosis of ADSC remains a problem in up to 20% of small biopsy cases, even using immunohistochemistry [23, 27]. A recent study reported by Zhang et al. showed that 38.8% (26/67) of post-operatively pathologically proven ADSC patients were pre-operatively misdiagnosed as SqCC and 29.8% (20/67) were misdiagnosed as poorly differentiated SqCC via biopsy or cytology specimens [28]. In this study, we found *EGFR* mutations are detected in 8.8% of small biopsy-diagnosed SqCC or NSCC favor SqCC, and significantly associated with never smokers and a diagnosis of NSCC favor SqCC. It has been reported that the incidence of *EGFR* mutations in Asian SqCC is less than 3% [29]. The frequency of *EGFR* mutations in our cases is higher than that reported in SqCC, suggesting that some of these small biopsy cases may not be single-histology SqCC. Indeed, three of 4 (75%) *EGFR* mutation-positive cases in our study are subsequently diagnosed as ADSC, suggesting that most of EGFR-mutated SqCC diagnosed from small biopsies may result from undersampling of ADSC.

The limitation of this study is that this is a retrospective study and patients who are diagnosed as SqCC in small biopsies would not receive EGFR-TKI treatment according to current guidelines, therefore, no treatment results are available for analysis. Moreover, due to the rarity of ADSC and its unfeasibility in small biopsy diagnosis, data on the efficacy of EGFR-TKI in ADSC patients is limited. Although there were only a limited number of cases published, some studies demonstrated that using EGFR-TKIs is an effective treatment for patients with EGFR-mutated ADSC (Table 3) [17, 28, 30]. Song et al. showed that the progression-free survival of ADSC patients with EGFR-TKIs treatment was 4.3 months which is comparable to 4.2 months of ADC. They also revealed higher efficacy of EGFR-TKIs in ADSC patients with *EGFR* mutations compared to those without *EGFR* mutations. In Fan et al. and Zhang et al. studies, EGFR-TKIs were only applied to *EGFR* activating mutation-positive patients, and the average of disease control rate and overall survival were 75.4% and 41 months. Zhang et al. also showed that ADSC patients harboring *EGFR*-sensitizing mutations who were treated with EGFR-TKIs had a significantly better prognosis than those receiving chemotherapy or chemoradiotherapy alone [28]. However, these studies only included patients with clinical stage I to IIIA, and a prospective study toward efficacy of EGFR-TKIs in advanced stage ADSC patients needs to be further conducted in the future.

**Table 3:** Results from current studies of EGFR-TKI efficacy on ADSC

Study	No. of patients	Ethnicity	Stage		Gender		Smoking status		EGFR activating mutation		Type of TKI	DCR (%)	PFS (m)	OS (m)
			I-IIIA	≥ IIIB	Male	Female	No	Yes	Positive	Negative				
Song et al., 2013 [17]	49	Asia	N/A		53.1% (26/49)	46.9% (23/49)	26.5% (13/49)	73.4% (36/49)	33.30% (7/21)	66.7% (14/21)	Gefitinib/ Erotinib	65.3% (32/49)	4.3 (2.1 for EGFR-; 8.7 for EGFR+)	17.6
Fan et al., 2017 [30]	27	Asia	85.2% (23/27)	14.8% (4/27)	40.7% (11/27)	59.3% (16/27)	74.1% (20/27)	25.9% (7/27)	100% (27/27)	0% (0/27)	N/A	74.1% (20/27)	15	39
Zhang et al., 2018 [28]	148	Asia	100% (148/148)	0% (0/148)	70.7% (102/148)	29.3% (46/148)	42.6% (63/148)	57.4% (85/148)	20.2% (30/148)	79.8% (118/148)	Gefitinib/ Erotinib	76.7% (23/30)	N/A	43

*DCR* Disease control rate, *PFS* Progression free survival, *OS* Overall survival, *N/A* Not applicable, *TKI* Tyrosine kinase inhibitor, *EGFR*- *EGFR* activating mutation negative, *EGFR*+ *EGFR* activating mutation positive

Our multivariate analyses showed that never-smokers and NSCC favor SqCC, but not female gender, are independently associated with *EGFR* mutations. Lung cancer in never smokers has significant gender and geographic variations, occurring more frequently in female and in Asia. One possible explanation is that the majority of female NSCLC patients in Asian populations have no smoking history. Studies showed that the proportion of never smokers in female lung cancer cases are 83% in South Asia, while only 15% in the United States [31]. Moreover, lung cancer in Asian never smokers are often diagnosed as ADC that can be classified into different molecular subtypes based on oncogenic driver mutations. On the contrary, SqCC is more significantly associated with smokers than other subtypes of NSCLC [32]. From a molecular point of view, several studies showed that *EGFR* mutations are more prevalent in tumors of never smokers, while *TP53* and *KRAS* mutations are more frequently found in smokers [31, 33–35]. Taken together, in our study cohort, the clinicopathological characteristics of *EGFR* mutation-positive cases are similar to those of ADC, implying that some of these cases might be EGFR-mutated ADSC. Furthermore, our findings that *EGFR* mutations significantly associate with a diagnosis of NSCC favor SqCC, which is defined as poorly differentiated NSCLC without definite SqCC morphology features, also suggest these cases may possibly be ADSC.

## Conclusions

EGFR-TKI is a therapeutic option of EGFR-mutated ADSC which is often misdiagnosed as SqCC in small lung biopsies, resulting in absence of *EGFR* mutation testing. Our findings demonstrated the importance of *EGFR* mutation testing in small biopsy-diagnosed lung SqCC, especially in never smokers and patients whose diagnoses need to be assisted by immunohistochemistry.

## Data Availability

The datasets supporting the conclusions of this article are available from the corresponding author on reasonable request.

## Abbreviations

ADC: Adenocarcinoma
ADSC: Adenosquamous carcinoma
ALK: Anaplastic lymphoma kinase
CAP/IASLC/AMP: The College of American Pathologists (CAP), the International Association for the Study of Lung Cancer (IASLC), and the Association for Molecular Pathology (AMP)
EGFR: Epidermal growth factor receptor
EGFR-TKIs: Epidermal growth factor receptor tyrosine kinase inhibitors
IHC: Immunohistochemistry
LCC: Large cell carcinoma
NSCC: Non-small cell carcinoma
NSCLC: Non-small cell lung cancer
SCLC: Small cell lung cancer
SqCC: Squamous cell carcinoma
TTF-1: Thyroid transcription factor-1
WHO: World Health Organization
